# Sex Differences in Characteristics, Treatments, and In-hospital Outcomes of Patients Undergoing Coronary Angiography or Intervention

**DOI:** 10.3389/fcvm.2022.878566

**Published:** 2022-04-29

**Authors:** Shi-Qun Chen, Jin Liu, Yang Zhou, Zhi-Dong Huang, Yun Xie, Hao-Zhang Huang, Xiao-Ming Yan, Yong-Yi Xie, Peng-Fei Hao, Yan Liang, Shao-Hong Dong, Xiao-Yu Huang, Li-Ling Chen, Ning Tan, Yong Liu, Ji-Yan Chen

**Affiliations:** ^1^Department of Cardiology, Guangdong Provincial People's Hospital, Guangdong Cardiovascular Institute, Guangdong Academy of Medical Sciences, Guangzhou, China; ^2^Department of Guangdong Provincial Key Laboratory of Coronary Heart Disease Prevention, Guangdong Cardiovascular Institute, Guangdong Provincial People's Hospital, Guangdong Academy of Medical Sciences, Guangzhou, China; ^3^The Second School of Clinical Medicine, Southern Medical University, Guangzhou, China; ^4^Department of Information Technology, Guangdong Provincial People's Hospital, Guangdong Academy of Medical Sciences, Guangzhou, China; ^5^Department of Cardiology, Maoming People's Hospital, Maoming, China; ^6^Department of Cardiology, Shenzhen People's Hospital, Shenzhen, China; ^7^Department of Cardiology, Yangjiang People's Hospital, Yangjiang, China; ^8^Department of Cardiology, Longyan First Affiliated Hospital of Fujian Medical University, Longyan, China; ^9^Guangdong Provincial People's Hospital, School of Medicine, South China University of Technology, Guangzhou, China

**Keywords:** outcome, coronary artery disease, percutaneous coronary intervention, coronary angiography, sex differences

## Abstract

**Background:**

Whether women have a higher risk of adverse events compared with men following coronary angiography (CAG) and percutaneous coronary intervention (PCI) remains controversial. We aimed to investigate the sex differences in characteristics, treatments and outcomes among patients undergoing CAG and PCI in a large Chinese cohort.

**Methods:**

We analyzed patients undergoing CAG and/or PCI in this multi-center registry cohort study Cardiorenal ImprovemeNt II (CIN-II) in 5 Chinese tertiary hospitals from 2007 to 2020. Clinical characteristics, treatment (discharge medication and PCI) and in-hospital outcomes (mortality and major bleeding) were compared between women and men.

**Results:**

Totally 141,459 patients underwent CAG (44,362 [31.4%] women), of which 69,345 patients underwent PCI (15,376 [22.2%] women). Women were older (64.4 vs. 60.8 years), had more chronic comorbidities and lower PCI rate for stable coronary artery disease (CAD) than men (52.8 vs. 64.2%). Women received less CAG and PCI procedures. Among women undergoing PCI they received similar discharge medication treatment. In addition, women undergoing PCI had mildly lower rate of major bleeding (0.2 vs. 0.3%, *P* = 0.033) but higher in-hospital mortality (1.2 vs. 0.8%, *P* < 0.001). After adjustment, women had a higher risk in the major bleeding (adjusted odds ratio, 2.04 [95% CI: 1.07 to 3.62]), and the in-hospital mortality (adjusted odds ratio, 1.87 [95% CI: 1.36 to 2.56]).

**Conclusion:**

Among our Chinese cohort, women are older with more chronic comorbidities, receiving less PCI procedure and similar discharge medication treatment. Women have nearly 90% higher risk of in-hospital mortality and over 1-fold increased risk of major bleeding after PCI compared with men.

## Introduction

Coronary artery disease (CAD) is a major cause of death and disability among both women and men globally (1, 2). Coronary angiography (CAG) is the reference standard for CAD diagnosis and percutaneous coronary intervention (PCI) remain the major revascularization strategy for patients with CAD, with an estimated five million procedures performed worldwide each year (3–7). Previous studies reported women undergoing PCI were older, suffered from more comorbidities, and had fewer chance to undergo PCI or other bypass treatments compared with men (8–10). However, some controversies exist in whether women or men have a better outcome among patients undergoing CAG and/or PCI in different countries (7, 10–13). Notably, even within the same country, the prognosis of men and women undergoing CAG and/or PCI is not uniform, which may be due to the difference of the ethnic and the regional medical conditions (14–16).

In China, the volumes of coronary catheterization increased continuously and were up to one million per year in 2020, becoming the largest annual volumes of coronary catheterization worldwide (17). However, there is limited real-world study to estimate sex differences in a large sample of patients undergoing catheterization with/without PCI in China.

This study included 141,459 CAG and PCI cases within the five large hospitals (two urban and three rural hospitals) from 2007 to 2020, and was objective to assess sex differences in patient characteristics, treatment and outcomes of Chinese patients undergoing catheterization with/without PCI.

## Methods

### Study Design and Population

**Figure 1:**
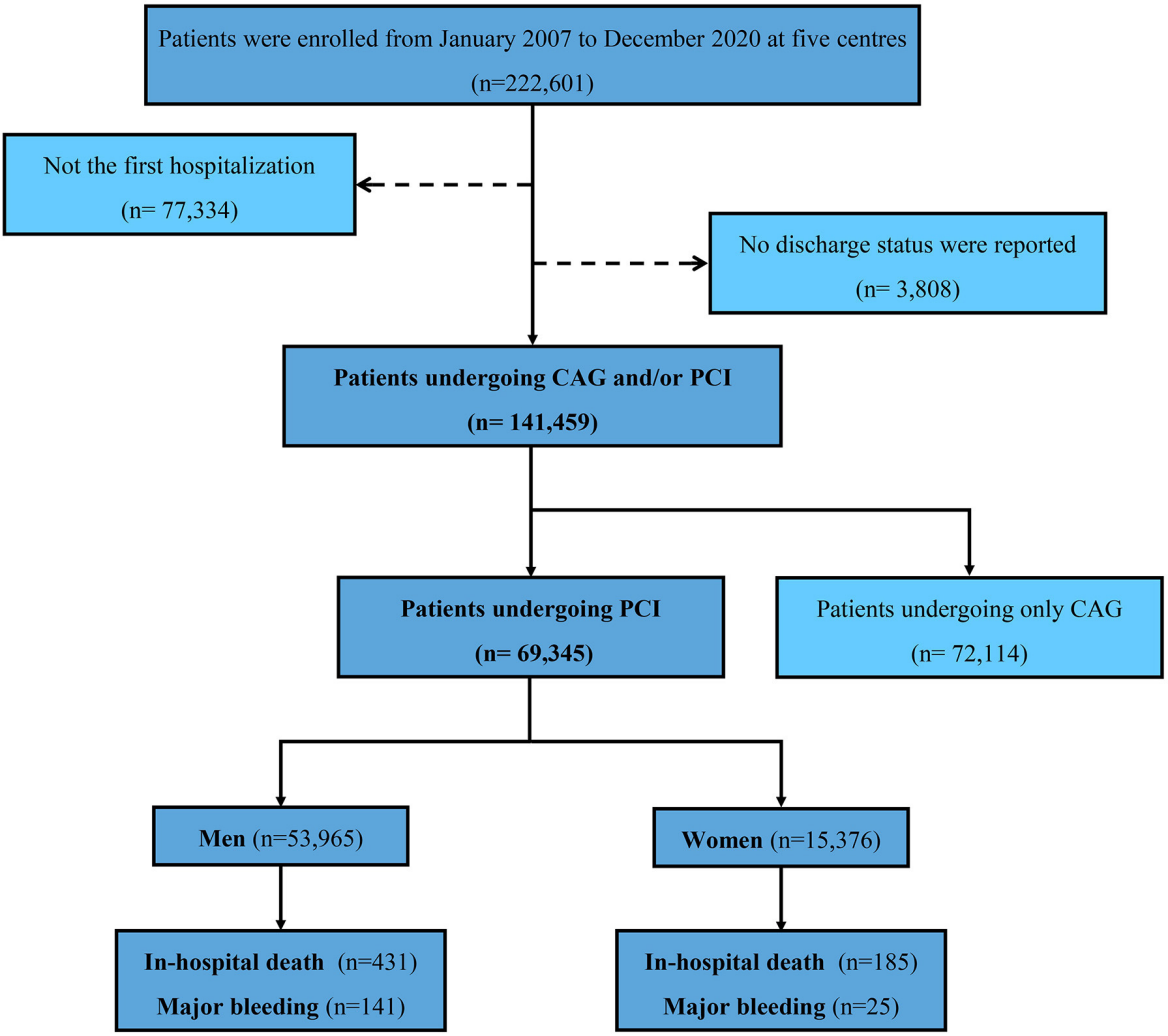
Flow diagram.

This multi-center, retrospective study was based on the registry of Cardiorenal ImprovemeNt II (CIN-II, NCT05050877) cohort from January 2000 to December 2020 in five south Chinese regional central tertiary teaching hospitals (two urban and three rural regions, see in Supplemental Table 1). Considering the time consistency among different centers, we included five centers covering patients from 2007 to 2020 undergoing CAG and/or PCI. Patients not hospitalized for CAG for the first time were excluded, as well as patients with missing the discharge status. Finally, 141,459 participants in CIN-II were enrolled (see Figure 1).

The study protocol was approved by the GDPH ethics committee (No.GDREC2019-555H-2), all participating sites received institutional review board approval from their own ethics committees, and the study was performed according to the declaration of Helsinki. Patients' informed consent was also not required for this study by the all-participating hospital's Ethics Committee.

### Data Collection

Data was collected from the Electronic Clinical Management System (ECMS) for all participant hospitals. The data was mainly included from six sections: demographics, discharge diagnosis, laboratory examinations, treatment (procedure and discharge medication), and discharge status. Clinical data was processed and imported into a relational database that can be queried using R, version 4.0.3 software (R Foundation for Statistical Computing, Vienna, Austria).

Senior cardiologists were responsible for the data quality control and periodical database verification. Finally, the accuracy of diagnosis and other variables underwent random on-site audits, with an error rate of <2% for 5,000 patients.

### Definitions and Variables

CAD was confirmed by CAG and discriminated according to the 10th Revision Codes of the International Classification of Diseases (ICD-10; I20.xx–I25.xx, I50.00001 and I91.40001 et al.). Congestive heart failure (CHF) was defined as the discharge diagnosis and New York Heart Association class >2 or Killip class >1. Chronic kidney disease (CKD) was defined as the discharge diagnosis and estimated glomerular filtration rate (eGFR) ≤ 60 mL/min/1.73 m^2^ using the Modification of Diet in Renal Disease (MDRD) formula (18). Hyperlipemia was defined according to 2016 European Society of Cardiology guidelines (19). Diabetes mellitus (DM) was accessed according to the discharge diagnosis, or HbA1c >6.5%, or treatment with a hypoglycemic agent or insulin. Hypertension (HT) was defined according to the 10th Revision Codes of the International Classification of Diseases I10.xxx-I12.xxx, I15.xxx and I67.400). Stable CAD was defined as CAD excluding acute myocardial infarction (AMI), ST-segment elevation myocardial infarction (STEMI), unstable angina or stable angina.

The key variables included demographic characteristics (age, sex, medical insurance type, standardized hospitalization cost), medical comorbidities (CAD, HT, DM, CHF, CKD, hyperlipemia and stoke), laboratory examinations (hemoglobin, serum creatine, low-density cholesterol [LDL-C] and high-density cholesterol [HDL-C], etc), procedural information (PCI, drug-eluting stent [DES], bare metal stent [BMS], intra-aortic balloon pump [IABP], rotational atherectomy, intravascular ultrasound [IVUS], optical coherence tomography [OCT], fractional flow reserve [FFR]), discharge medication (Dual antiplatelet therapy (Aspirin/ Clopidogrel or Ticagrelor), renin-angiotensin-aldosterone system [RAAS] blocker and beta-blockers, Statins). Patients who underwent CAG or PCI were performed following standard clinical practice guidelines (20–22).

### Study Outcomes

In-hospital outcomes were analyzed in patients undergoing PCI. The primary outcome is all-cause in-hospital mortality, and the secondary outcome is the major bleeding. Major bleeding was defined based on the Thrombolysis in Myocardial Infarction (TIMI) bleeding criteria (Clinically overt signs of hemorrhage associated with a drop in hemoglobin over 5 g/dL) (23).

### Statistical Analysis

We described demographics, treatment (procedure), and outcomes of patients undergoing CAG and/or PCI from 2007 to 2020. The continuous variables were described by means ± standard deviation (SD) or median [interquartile ranges (IQRs)], and categorical variables were presented as counts and proportions. Student *t*-test, Kruskal-Wallis and chi-square tests were used to compare the differences between groups as appropriate. Descriptive statistic for categorical variables is reported as numbers (percentages).

Multivariable logistic regression analyses were performed to determine the relationship between sex and study outcomes. Variables based on clinical plausibility were included in the multivariable models (age, AMI, HT, DM, stroke, CHF, CKD, atrial fibrillation, hyperlipemia, prior MI, prior PCI, hemoglobin and dual-antiplatelet therapy [DAPT]). The odds ratio (OR) 95% with confidence interval (CI) and *P*-values were calculated. A two-sided *P*-value <0.05 indicated significance for all analyses. All statistical analyses were performed using R version 4.0.3 software.

## Results

### Characteristics of Patients Undergoing CAG With or Without PCI

Our study population included 141,459 patients undergoing CAG (44,362 [31.4%] women, mean age: 64.4 vs. 60.8 years), of which 69,345 patients undergoing PCI (15,376 [22.2%] women, mean age: 67.4 vs. 61.2 years) in 5 Chinese tertiary hospitals from 2007 to 2020. Totally 70.1% patients (*n* = 96,687) suffered from CAD, 19.9% patients (*n* = 27,404) had AMI, 50.8% patients (*n* = 70,078) were complicated with HT, 30.0% patients (*n* = 41,839) had DM and 16.9% patients (*n* = 23,492) were combined with CKD. In addition, 32.0% patients (*n* = 45,231) had stable CAD.

Compared with men, women received less CAG and/or PCI procedures, and women undergoing CAG and/or PCI were older. Women undergoing CAG tended to suffer from chronic comorbidities including HT (55.5 vs. 48.7%, *P* < 0.001), DM (31.9 vs. 29.1%, *P* < 0.001) and CKD (17.2 vs. 16.7%, *P* = 0.028), while acute comorbidities like AMI (12.1 vs. 23.4%, *P* < 0.001) were less than men. In addition, the proportion of women with a history of PCI (4.4 vs. 8.2%, *P* < 0.001), MI (2.2 vs. 5.3%, *P* < 0.001) and coronary artery bypass graft (CABG) (0.2 vs. 0.4%, *P* < 0.001) was significantly less than that of men. The detailed patients' clinical characteristics are listed in Table 1.

**Table 1:** Demographic and clinical characteristics of patients undergoing CAG and/or PCI from 2007 to 2020.

**Characteristic**	**Overall**	**Men**	**Women**	***P*-value**
	***N* = 141,459**	***N* = 97,088**	***N* = 44,362**	
**Demographic characteristics**				
Age, year (mean)	61.9 (11.1)	60.8 (11.3)	64.4 (10.0)	<0.001
**Age group**, ***n*** **(%)**				<0.001
≤ 60	61,873 (44.0)	46,358 (48.0)	15,510 (35.2)	
>60 and ≤ 75	62,693 (44.6)	40,460 (41.9)	22,229 (50.4)	
>75	16,027 (11.4)	9,698 (10.0)	6,329 (14.4)	
Insurance coverage, *n* (%)	1,18,849 (85.1)	81,247 (84.7)	37,593 (85.9)	<0.001
Hospitalization cost, ¥ median (IQR)	40324.8 [10213.9, 66532.2]	42757.8 [11952.1, 68163.8]	31866.7 [8797.6, 62208.6]	<0.001
**Comorbidities**				
Coronary artery disease, *n* (%)	96,687 (70.1)	72,714 (76.6)	23,968 (55.8)	<0.001
AMI, *n* (%)	27,404 (19.9)	22,183 (23.4)	5,217 (12.1)	<0.001
Hypertension, *n* (%)	70,078 (50.8)	46,236 (48.7)	23,839 (55.5)	<0.001
Diabetes mellitus, *n* (%)	41,839 (30.0)	27,951 (29.1)	13,886 (31.9)	<0.001
Anemia, *n* (%)	29,373 (23.6)	19,857 (23.2)	9,516 (24.5)	<0.001
Congestive heart failure, *n* (%)	21,091 (15.3)	14,759 (15.5)	6,330 (14.7)	<0.001
Chronic kidney disease, *n* (%)	23,492 (16.9)	16,019 (16.7)	7,473 (17.2)	0.028
Atrial fibrillation, *n* (%)	9,585 (7.0)	5,626 (5.9)	3,958 (9.2)	<0.001
Stroke, *n* (%)	8,001 (5.8)	5,529 (5.8)	2,472 (5.8)	0.622
Hyperlipemia, *n* (%)	74,695 (53.6)	54,256 (56.6)	20,436 (47.0)	<0.001
Prior PCI, *n* (%)	9,700 (7.0)	7,796 (8.2)	1,904 (4.4)	<0.001
Prior MI, *n* (%)	5,991 (4.3)	5,064 (5.3)	927 (2.2)	<0.001
Prior CABG, *n* (%)	474 (0.3)	399 (0.4)	75 (0.2)	<0.001
**Laboratory tests**				
eGFR, mL/min/1.73 m^2^	80.1 (25.2)	80.2 (25.7)	79.9 (24.2)	0.052
Hemoglobin, g/L	134.2 (17.4)	138.4 (16.9)	125.0 (14.7)	<0.001
Proir SCr, umol/L	1.0 (1.4)	1.1 (1.5)	0.9 (1.2)	<0.001
LDLC, mmol/L	2.9 (1.0)	2.8 (1.0)	3.0 (1.0)	<0.001
HDLC, mmol/L	1.1 (0.3)	1.0 (0.3)	1.2 (0.3)	<0.001
**Discharge medications**				
Dual-antiplatelet therapy, *n* (%)	71,752 (60.7)	54,873 (67.4)	16,877 (45.8)	<0.001
RAAS blocker, *n* (%)	73,577 (62.2)	52,580 (64.6)	20,995 (57.0)	<0.001
Drug β blocker, *n* (%)	83,062 (70.3)	59,558 (73.2)	23,501 (63.8)	<0.001
Statins, *n* (%)	98,283 (83.1)	70,621 (86.8)	27,657 (75.1)	<0.001

*AMI, Acute myocardial infarction; Prior PCI, Prior percutaneous coronary intervention; Prior MI, Prior myocardial infarction; Prior CABG, Prior coronary artery bypass graft; eGFR, estimated glomerular filtration rate; Prior SCr, Prior serum creatinine; LDLC, Low density liptein cholesterol; HDLC, High density liptein cholesterol; RAAS, Renin-angiotensin-aldosterone system*.

### PCI Patients' Characteristics Among Men and Women

**Figure 2:**
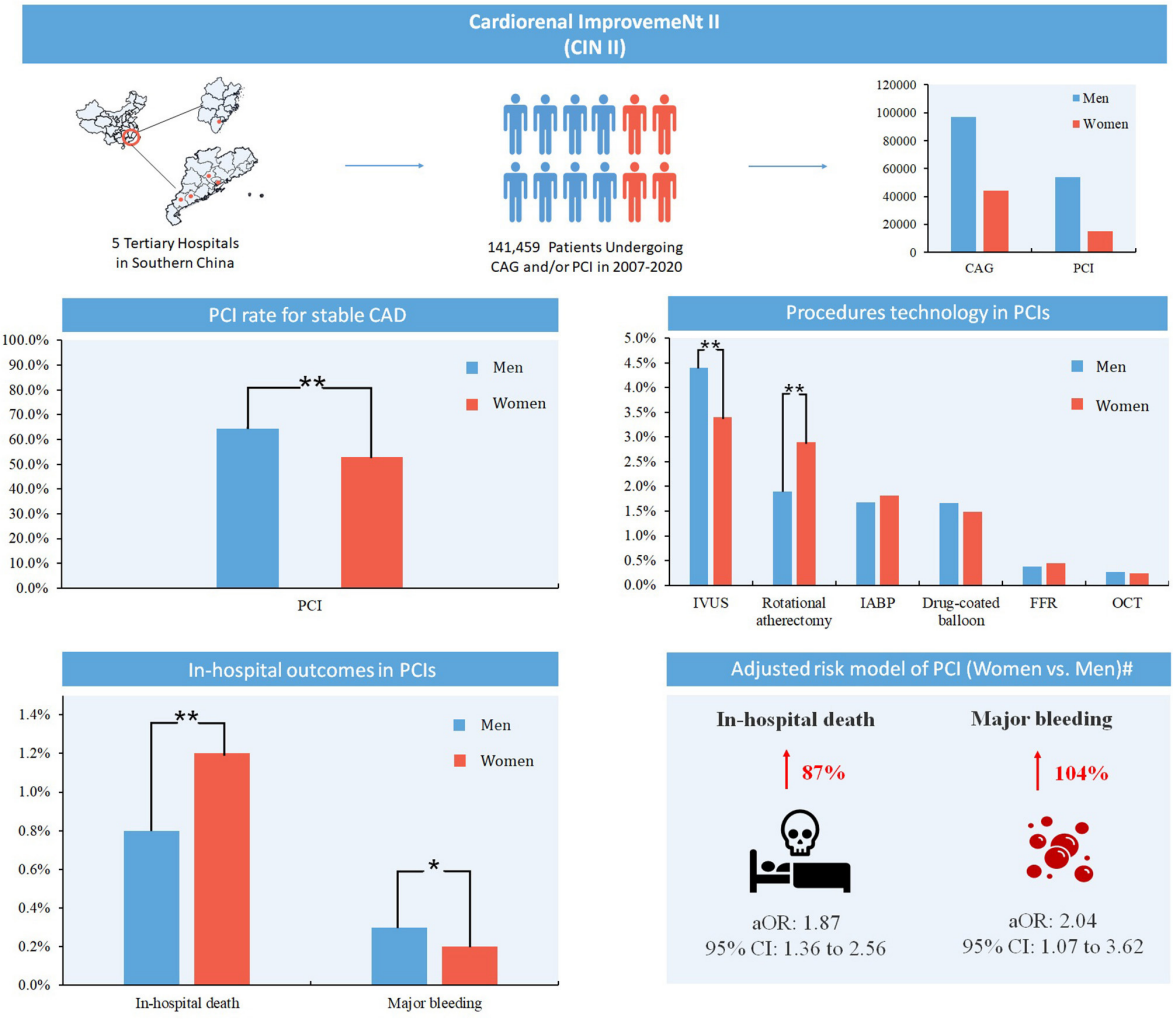
Centre illustrations. CAG, coronary angiography; PCI, percutaneous coronary intervention; CAD, coronary artery disease; IVUS, intravascular ultrasound; IABP, intra-aortic balloon pump; FFR, fractional flow reserve; OCT, optical coherence tomography. **: *P* < 0.001; *: *P* < 0.05. #: Adjusted for age, acute myocardial infarction, hypertension, diabetes mellitus, congestive heart failure, chronic kidney disease, atrial fibrillation, stroke, hyperlipemia, prior myocardial infarction, prior percutaneous coronary intervention, hemoglobin and dual-antiplatelet therapy.

Similar to patients undergoing CAG, women undergoing PCI had more chronic comorbidities including HT (67.7 vs. 51.1%), DM (44.1 vs. 33.6%), and CKD (24.0 vs. 18.1%), while acute comorbidities like AMI (28.3 vs. 36.6%) were less than men. Furthermore, the proportion of women with a history of PCI (7.0 vs. 9.2%), MI (3.8 vs. 6.5%) was significantly less than that of men. (All *P*-values <0.001) (Table 2). In addition, the PCI rate for stable CAD patients was significantly lower in women than in men (52.8 vs. 64.2%, *P* < 0.001) (Figure 2. Centre illustrations).

**Table 2:** Demographic and clinical characteristics of patients undergoing PCI from 2007 to 2020.

**Characteristic**	**Overall**	**Men**	**Women**	***P*-value**
	***N* = 69,345**	***N* = 53,965**	***N* = 15,376**	
**Demographic characteristics**
Age, year (mean)	62.6 (11.2)	61.2 (11.2)	67.4 (9.6)	<0.001
**Age group**, ***n*** **(%)**				<0.001
≤ 60	28,418 (41.2)	24,963 (46.5)	3,453 (22.6)	
>60 and ≤ 75	31,572 (45.8)	22,944 (42.7)	8,626 (56.4)	
>75	8,975 (13.0)	5,773 (10.8)	3,202 (21.0)	
Insurance coverage, *n* (%)	58,189 (84.9)	45,190 (84.7)	12,995 (85.6)	0.004
Hospitalization cost, ¥ median (IQR)	53698.6 [41041.6, 74501.2]	54403.9 [41374.8, 75652.8]	51410.9 [40037.4, 70707.8]	<0.001
**Comorbidities**
AMI, *n* (%)	24,086 (34.7)	19,728 (36.6)	4,354 (28.3)	<0.001
Hypertension, *n* (%)	37,994 (54.8)	27,584 (51.1)	10,409 (67.7)	<0.001
Diabetes mellitus, *n* (%)	24,925 (35.9)	18,135 (33.6)	6,788 (44.1)	<0.001
Anemia, *n* (%)	16,533 (26.7)	12,284 (25.6)	4,249 (30.9)	<0.001
Congestive heart failure, *n* (%)	12,814 (18.5)	9,705 (18.0)	3,107 (20.2)	<0.001
Chronic kidney disease, *n* (%)	13,463 (19.4)	9,780 (18.1)	3,683 (24.0)	<0.001
Atrial fibrillation, *n* (%)	2,418 (3.5)	1,774 (3.3)	643 (4.2)	<0.001
Stroke, *n* (%)	4,148 (6.0)	3,163 (5.9)	985 (6.4)	0.013
Hyperlipemia, *n* (%)	4,0794 (58.8)	3,2578 (60.4)	8,215 (53.4)	<0.001
Prior PCI, *n* (%)	6,008 (8.7)	4,939 (9.2)	1,069 (7.0)	<0.001
Prior MI, *n* (%)	4,086 (5.9)	3,505 (6.5)	581 (3.8)	<0.001
Prior CABG, *n* (%)	261 (0.4)	210 (0.4)	51 (0.3)	0.341
**Laboratory tests**
eGFR, mL/min/1.73 m^2^	78.4 (26.3)	79.4 (26.3)	75.0 (25.8)	<0.001
Hemoglobin, g/L	134.2 (17.7)	137.6 (17.0)	122.7 (15.2)	<0.001
Proir SCr, umol/L	1.1 (1.5)	1.1 (1.6)	0.9 (1.0)	<0.001
LDLC, mmol/L	2.9 (1.0)	2.9 (1.0)	3.1 (1.1)	<0.001
HDLC, mmol/L	1.0 (0.3)	1.0 (0.3)	1.1 (0.3)	<0.001
**Discharge medications**
Dual-antiplatelet therapy, *n* (%)	57,215 (95.5)	44,667 (95.7)	12,546 (94.9)	<0.001
RAAS blocker, *n* (%)	44,380 (74.1)	34,438 (73.7)	9,941 (75.2)	0.001
Drug β blocker, *n* (%)	49,907 (83.3)	38,924 (83.4)	10,981 (83.1)	0.481
Statins, *n* (%)	58,562 (97.7)	45,682 (97.8)	12,877 (97.4)	0.009

*AMI, Acute myocardial infarction; Prior PCI, Prior percutaneous coronary intervention; Prior MI, Prior myocardial infarction; Prior CABG, Prior coronary artery bypass graft; eGFR, estimated glomerular filtration rate; Prior SCr, Prior serum creatinine; LDLC, Low density liptein cholesterol; HDLC, High density liptein cholesterol; RAAS, Renin-angiotensin-aldosterone system*.

### PCI Procedure and Discharge Medication Information

From 2007 to 2020, women undergoing PCI received less IVUS (3.4 vs. 4.4%, *P* < 0.001), but more rotational atherectomy (2.9 vs. 1.9%, *P* < 0.001), while no significant differences were observed in other procedures (e.g., drug-coated balloon, OCT, FFR and IABP) (Table 2). In addition, among women undergoing PCI, the use of RAAS blocker was higher (75.2 vs. 73.7%, *P* = 0.001), but the use of statins (97.4 vs. 97.8%, *P* = 0.009) and DAPT (94.9 vs. 95.7%, *P* < 0.001) was moderately lower. Among patients undergoing PCI, women received PCI procedures mainly for the treatment of stable CAD, while men for the treatment of AMI.

### Study Outcomes

Among patients undergoing PCI, in-hospital mortality was higher in women than men (1.2 vs. 0.8%, *P* < 0.001). After adjusting for confounding factors (age, AMI, HT, DM, stroke, CHF, CKD, atrial fibrillation, hyperlipemia, prior MI, prior PCI, hemoglobin and DAPT), women undergoing PCI still had higher in-hospital mortality (aOR [95% CI]: 1.87 [95% CI: 1.36 to 2.56], *P* < 0.001). In addition, the rate of major bleeding was mildly lower in women (0.2 vs. 0.3%, *P* = 0.033), but after adjusting for confounders, women had a higher risk of major bleeding (aOR [95% CI]: 2.04 [95% CI: 1.07 to 3.62], *P* = 0.022) (Table 3).

**Table 3:** Multivariate risk regression model of PCI.

**Variables**	**In-hospital death**		**Major bleeding**	
	**OR (95% CI)**	***P*-value**	**OR (95% CI)**	***P*-value**
Women *vs*. Men	1.87 (1.36–2.56)	<0.001	2.04 (1.07–3.62)	0.022
Age, year	1.03 (1.02–1.04)	<0.001	1.00 (0.99–1.23)	0.664
AMI	5.32 (3.93–7.27)	<0.001	2.33 (1.44–3.84)	<0.001
Hypertension	0.77 (0.58–1.01)	0.057	0.81 (0.53–1.23)	0.325
Diabetes mellitus	1.13 (0.85–1.48)	0.403	1.52 (1.00–2.30)	0.047
CHF	0.98 (0.71–1.32)	0.874	4.16 (2.63–6.68)	<0.001
CKD	1.02 (0.73–1.41)	0.908	3.03 (1.93–4.72)	<0.001
Atrial fibrillation	1.40 (0.77–2.36)	0.235	2.25 (1.09–4.23)	0.018
Stroke	2.98 (2.02–4.29)	<0.001	1.91 (0.91–3.64)	0.065
Hyperlipemia	0.65 (0.50–0.85)	0.002	1.02 (0.67–1.58)	0.917
Prior MI	0.78 (0.30–1.64)	0.553	0.18 (0.01–0.87)	0.095
Prior PCI	1.29 (0.72–2.13)	0.357	0.89 (0.33–2.01)	0.801
Hemoglobin	1.01 (1.00–1.02)	0.007	1.10 (1.08–1.11)	<0.001
DAPT	0.62 (0.39–1.07)	0.064	0.35 (0.18–0.74)	0.003

*AMI, Acute myocardial infarction; CHF, Congestive heart failure; CKD, Chronic kidney disease; Prior MI, Prior myocardial infarction; DAPT, Dual-antiplatelet therapy*.

## Discussion

To our knowledge, this study is the largest multicentre study in China to evaluate the sex differences of patients undergoing coronary intervention. We reported the sex difference in patients undergoing CAG and/or PCI from 2007 to 2020, including characteristic, treatment and outcomes. Compared with men, women were older, received less CAG and/or PCI procedures, had more chronic comorbidities and received fewer interventions and drug therapies. Women also had higher risk of in-hospital mortality and major bleeding than men.

In our study, women were older, and more suffered from chronic comorbidities including CHF, CKD, and DM, while men more suffered from acute comorbidities like AMI. In addition, the proportion of women with a history of PCI, MI and CABG was significantly less than that of men. Findings of our study is similar to a meta-analysis: among 32,877 patients undergoing PCI, women are older, more likely to suffer from HT and DM, and less frequent history of surgical or percutaneous revascularization compared with men (8). Furthermore, our study found that the PCI rate for stable CAD is significantly lower in women than in men (52.8 vs. 64.2%), which may suggest the overdiagnosis in men with CAD or the underestimation of the severity of stable CAD in women patients.

During the study period, we found that women received fewer CAG and PCI procedures than men. Women also received fewer drug therapies, including DAPT, β blocker and statins. In addition, the hospitalization cost for women was much lower than for men, possibly because women had fewer chance to receive PCI or other bypass therapy compared with men. Khera et al. have illustrated that younger women are less likely to receive revascularization for STEMI and have higher in-hospital mortality than younger men (9). This study was limited to younger patients with STEMI aged 18 to 59 years, and our study included a wider age range of patients, making the data more representative. In addition, our study found that the rate of women undergoing rotational atherectomy is much high than men. In fact, the high rate of rotational atherectomy demonstrates the severity of the CAD in women (24).

The outcomes of CAD patients undergoing PCI also deserve attention. In our study, we found the in-hospital mortality of woman was higher than that of man. Gevaert et al. evaluated differences in in-hospital mortality between men and women with TIMI risk score variables and found a crude in-hospital mortality rate of 10.1% in women and 4.9% in men among a prospective, observational study of 8,073 STEMI patients (25). But this study included consecutive pPCI-treated patients with STEMI, which makes the mortality of their study higher than ours. A retrospective cohort of 134,501 older (≥75 years) AMI-CS admissions also found female sex an independent predictor of higher in-hospital mortality (56.6 vs. 55.1%) (26). It may be because patients in their study are older with more comorbidities, which makes their mortality much higher than ours. Furthermore, we found the risk of major bleeding was also higher in women among patients undergoing PCI after adjusting for confounders. Contrary to our study, recent study from Korea demonstrated that women with AMI is not associated with an increased risk of major bleeding. The result of this study differs from ours in that they reported bleeding outcomes separately, as 1-year thrombolysis in myocardial infarction (TIMI) major and minor bleeding events (27). Similar to our study, Yu et al. (28) demonstrated that the risk of major bleeding was 1.8-fold higher in women in the Harmonizing Outcomes with Revascularization and Stents in Acute Myocardial Infarction trial. But this study defined the major bleeding as overt bleeding with hemoglobin decreasing >3 g/dl or >4 g/dl with no overt source of bleeding. Kodaira et al. also showed that women has a higher risk of bleeding in 2,494 acute coronary syndrome (ACS) patients undergoing PCI (29). The bleeding events of this study occurred in patients after discharge and not all bleeding events were more common in women such as the hemorrhagic stroke (26.6 vs. 29.6%). Our data of major bleeding are in hospital, and the data after discharge still requires follow-up.

Findings also suggested that the hospitalization cost among women patients undergoing PCI was lower, possibly because women had less chance to receive intervention therapies or were unwilling to receive them, which significantly increased their mortality after coronary intervention. It is necessary to carry out early risk assessment and treatment for them, including CAG diagnosis and PCI treatment. In addition, the indications for PCI need to be evaluated among patients with stable CAD, and women undergoing CAG or PCI should be considered as a focus group for perioperative major bleeding prevention. Attention should also be paid to the prognosis of old patients with many comorbidities undergoing PCI. More studies are needed in the future to improve outcomes in women with CAD after PCI, including the risk prevention strategy for all-cause mortality.

### Limitations

This study has several limitations. First, this is a retrospective study, and there may be data gaps including the body mass index (BMI) and smoking history of patients. But our study has large data, and systematically evaluated sex differences among patients undergoing CAG and PCI. Second, our study was conducted in teaching hospitals in southern China, and cannot be extended to patients undergoing coronary intervention throughout China for the time being. However, the study population was distributed in urban and rural areas, which was representative to some extent. Third, the volume of men and women included in our study was uneven, but based on real-world data, more men than women underwent coronary intervention. Additionally, our study did not subdivide all-cause mortality and major bleeding, and lacked long-term follow-up, but the main outcomes of coronary intervention were in-hospital mortality and major bleeding, which could reflect the medical quality and sex differences in coronary intervention.

## Conclusion

In our study, women were older with more chronic comorbidities, receiving less PCI procedure and similar discharge medication treatment compared with men. Women had nearly 90% higher risk of in-hospital mortality and over 1-fold increased risk of major bleeding after PCI. It suggests that clinicians should carry out early risk assessment and treatment for women patients, and consider women undergoing CAG or PCI as a focus group for perioperative major bleeding prevention.

## Data Availability Statement

The original contributions presented in the study are included in the article/Supplementary Material, further inquiries can be directed to the corresponding author/s.

## Ethics Statement

The studies involving human participants were reviewed and approved by Guangdong Provincial People's Hospital Ethics Committee. Written informed consent was not required for this study, in accordance with the local legislation and institutional requirements.

## Disclosure

This study was approved by Guangdong Provincial People's Hospital ethics committee and the study was performed according to the declaration of Helsinki. Informed consent was not required for this study by the Guangdong Provincial People's Hospital Ethics Committee.

## Author Contributions

YL, S-QC, JL, and YZ: research idea and study design. YX, X-MY, H-ZH, Y-YX, P-FH, YL, S-HD, X-YH, and L-LC: data acquisition. S-QC and JL: data analysis/interpretation. Z-DH: statistical analysis. J-YC, YL, and NT: supervision and mentorship. Each author contributed important intellectual content during manuscript drafting or revision and accepts accountability for the overall work by ensuring that questions pertaining to the accuracy or integrity of any portion of the work are appropriately investigated and resolved.

## Funding

This work was supported by grants from Beijing Lisheng Cardiovascular Health Foundation (LHJJ20141751); Guangdong Provincial Science and Technology Project (2020B1111170011), and National Science Foundation of China (81670339, 82070360). The work was not funded by any industry sponsors.

## Conflict of Interest

The authors declare that the research was conducted in the absence of any commercial or financial relationships that could be construed as a potential conflict of interest.

## Publisher's Note

All claims expressed in this article are solely those of the authors and do not necessarily represent those of their affiliated organizations, or those of the publisher, the editors and the reviewers. Any product that may be evaluated in this article, or claim that may be made by its manufacturer, is not guaranteed or endorsed by the publisher.
