# Supplementary material for: Sex Differences in Characteristics, Treatments, and In-hospital Outcomes of Patients Undergoing Coronary Angiography or Intervention
Source: Front Cardiovasc Med. 2022 Apr 29;9:878566. doi: 10.3389/fcvm.2022.878566 (PMC9106109; doi:10.3389/fcvm.2022.878566)
Supplement: Supplementary file 1 [file Data_Sheet_1.docx]

**Supplemental Table S1 Modules of data available in the CIN II database**

| **Class of data** | **Description** |
| --- | --- |
| Billing | Coded data recorded primarily for billing and administrative purposes. Includes Current Procedural Terminology (CPT) codes, Diagnosis-Related Group (DRG) codes, and International Classification of Diseases (ICD) codes. |
| Descriptive | Demographic detail, admission and discharge times, and dates of death. |
| Dictionary | Look-up tables for cross referencing concept identifiers (for example, International Classification of Diseases (ICD) codes) with associated labels. |
| Interventions | Procedures such as dialysis, imaging studies, and placement of lines. |
| Laboratory | Blood chemistry, hematology, urine analysis, and microbiology test results. |
| Medications | Administration records of intravenous medications and medication orders. |
| Notes | Free text notes such as provider progress notes and hospital discharge summaries. |
| Physiologic | Nurse-verified vital signs, approximately hourly (e.g., heart rate, blood pressure, respiratory rate). |
| Echocardiography | A painless test that uses sound waves to create moving pictures of your heart. The pictures show the size and shape of your heart. They also show how well your heart's chambers and valves are working. |
| Reports | Free text reports of electrocardiogram and imaging studies. |

**Supplemental Table S2 missing data in CAGs and PCIs**

| Characteristic | CAG | PCI |
| --- | --- | --- |
| Age, year (mean) | 866(0.61) | 380(0.55) |
| Age group, n(%) | 866(0.61) | 380(0.55) |
| Insurance coverage, n(%) | 1770(1.25) | 806(1.16) |
| Hospitalization cost, ￥median(IQR) | 13511(9.55) | 6733(9.71) |
| CAD, n(%) | 3585(2.53) | 0(0) |
| AMI, n(%) | 3585(2.53) | 0(0) |
| Hypertension, n(%) | 3585(2.53) | 0(0) |
| Diabetes mellitus, n(%) | 1963(1.39) | 0(0) |
| Anemia, n(%) | 17160(12.13) | 7507(10.83) |
| CHF, n(%) | 3585(2.53) | 0(0) |
| CKD, n(%) | 2247(1.59) | 0(0) |
| Atrial fibrillation, n(%) | 3585(2.53) | 0(0) |
| Stroke, n(%) | 3585(2.53) | 0(0) |
| Hyperlipemia, n(%) | 2049(1.45) | 0(0) |
| Prior PCI, n(%) | 3585(2.53) | 0(0) |
| Prior MI, n(%) | 3585(2.53) | 0(0) |
| Prior CABG, n(%) | 3585(2.53) | 0(0) |
| eGFR, mL/min/1.73m^2^ | 16361(11.57) | 6689(9.65) |
| HGB, g/L | 17042(12.05) | 7403(10.68) |
| Prior SCr, umol/L | 16156(11.42) | 6643(9.58) |
| LDLC, mmol/L | 24067(17.01) | 11349(16.37) |
| HDLC, mmol/L | 33509(23.69) | 15969(23.03) |
| Drug-coated balloon, n(%) | 0(0) | 0(0) |
| Rotational atherectomy, n(%) | 0(0) | 0(0) |
| IVUS, n(%) | 0(0) | 0(0) |
| OCT, n(%) | 0(0) | 0(0) |
| FFR, n(%) | 0(0) | 0(0) |
| IABP, n(%) | 0(0) | 0(0) |
| DAPT, n(%) | 23229(16.42) | 9428(13.6) |
| RAAS, n(%) | 23229(16.42) | 9428(13.6) |
| Drug β blocker, n(%) | 23229(16.42) | 9428(13.6) |
| Statins, n(%) | 23229(16.42) | 9428(13.6) |

CAD: Coronary artery disease; AMI: Acute myocardial infarction; HT: Hypertension; DM: Diabetes mellitus; CHF: Congestive heart failure; CKD: Chronic kidney disease; AF: Atrial fibrillation; Prior PCI: Prior percutaneous coronary intervention; Prior MI: Prior myocardial infarction; Prior CABG: Prior coronary artery bypass graft; eGFR EPI: estimated glomerular filtration rate epidemiology collaboration equation; HGB: Hemoglobin; Prior SCr: Prior serum creatinine; LDLC: Low density liptein cholesterol; HDLC: High density liptein cholesterol; IVUS: Intravascular ultrasound; OCT: Optical coherence tomography; FFR: Fractional flow reserve; IABP: Intra-aortic ballon pump; DAPT: Dual-antiplatelet therapy; RAAS: Renin-angiotensin-aldosterone system blocker.
